# Association between a history of mycobacterial infection and the risk of newly diagnosed Sjögren’s syndrome: A nationwide, population-based case-control study

**DOI:** 10.1371/journal.pone.0176549

**Published:** 2017-05-09

**Authors:** Wen-Cheng Chao, Ching-Heng Lin, Tsai-Ling Liao, Yi-Ming Chen, Der-Yuan Chen, Hsin-Hua Chen

**Affiliations:** 1 Department of Medical Research, Taichung Veterans General Hospital, Taichung, Taiwan; 2 Division of Chest Medicine, Department of Internal Medicine, Taichung Veterans General Hospital, Taichung, Taiwan; 3 Department of Business Administration, National Changhua University of Education, Changhua, Taiwan; 4 Institute of Biomedical Science and Rong Hsing Research Center for Translational Medicine, Chung-Hsing University, Taichung, Taiwan; 5 Division of Allergy, Immunology and Rheumatology, Department of Internal Medicine, Taichung Veterans General Hospital, Taichung, Taiwan; 6 School of Medicine, National Yang-Ming University, Taipei, Taiwan; 7 School of Medicine, Chung-Shan Medical University, Taichung, Taiwan; 8 Department of Medical Education, Taichung Veterans General Hospital, Taichung, Taiwan; 9 Institute of Public Health and Community Medicine Research Center, National Yang-Ming University, Taipei, Taiwan; Indian Institute of Technology Delhi, INDIA

## Abstract

**Objective:**

To address the association between a history of tuberculosis (TB) or nontuberculous mycobacterial (NTM) infection and the risk of newly diagnosed Sjögren’s syndrome (SS).

**Methods:**

Using a nationwide, population-based, claims dataset, and after excluding those who had rheumatoid arthritis or systemic lupus erythematosus, we identified 5,751 newly diagnosed SS cases during 2007–2012, and compared them to 86,265 non-SS controls matched (1:15) for age, sex, and the year of first SS diagnosis date. The association between the risk of incident SS and a history of mycobacterial infection, including TB and NTM, was quantified by calculating odds ratios (ORs) with 95% confidence intervals (CIs) using conditional logistic regression analysis after adjustment for Charlson comorbidity index (CCI) and bronchiectasis.

**Results:**

The mean age was 55±14 years, and the proportion of female gender was 87.8% in both newly diagnosed SS cases andnon-SS controls. An association was observed between NTM infection (OR, 11.24; 95% CI, 2.37–53.24) and incident SS, but not between TB infection and incident SS (OR, 1.29; 95% CI, 0.97–1.71) after adjustment for CCI and bronchiectasis. The association between NTM and SS risk was remarkably strong among those aged between 45 and 65 years (OR, 39.24; 95% CI, 3.97–387.75) and those without bronchiectasis (OR, 39.98; 95% CI, 3.83–376.92).

**Conclusion:**

The study reveals a significant association of newly diagnosed SS with a history NTM infection, especially among individuals aged 40–65 years or those without bronchiectasis.

## Introduction

Sjögren’s syndrome (SS) is estimated to affect approximately 1% of the general population and characterized to occur predominantlyin middle-aged women, with a female-to-male ratio of approximately 9:1 [1]. SS majorly presented with an insidious onset of dry eyes and dry mouth, which might be ignored by patients [2, 3]. Therefore, the early diagnosis of SS has been shown to be difficult. Currently, the pathogenesis of SS remains elusive. Environmental, genetic and hormonal contributors appear to be involved [4]. Mycobacterial infections,including tuberculosis (TB) and nontuberculous mycobacterial (NTM) infection, are characterized by a subacute clinical course with a dysregulated granulomatous inflammation [5] and have been implicated in the development of autoimmunity [6, 7]. Moreover, both SS and NTM infection predominantly affect middle-aged women, indicating potential shared mechanisms in these two diseases [8–10]. An increased incidence of TB has been reportedin subjects with SS [11]; however, whether TB or NTM infection is associated with the risk of SS is still unknown. The aim of this study is to investigate the association between mycobacterial infection and the risk of newly diagnosed SS using a population-based, longitudinal dataset.

## Methods

### Ethical statements

This study was approved by the Institutional Review Board of Taichung Veterans General Hospital Taiwan (IRB number: CE16251A). Informed consent was waived, given that the personal data obtained were anonymised before analysis.

### Study design and data source

Taiwan launched a single-payer National Health Insurance (NHI) programme on March 1, 1995. As of 2015, 99.6% of Taiwan’s population were enrolled [12]. The National Health Insurance Research Data (NHIRD) is the database of the programme, containing registration files and original claim data for reimbursement. The Bureau of NHI (BNHI) managed the NHIRD and released the data for research purpose. In this retrospective study, the ambulatory, inpatient and enrollment data from the 2003–2012 NHIRD were usedas discussed below to identify all patients with newly diagnosed SS. Moreover, in Taiwan, patients with certain major illnesses, such as cancer and certain autoimmune diseases, including SS, are issued a certificate of “catastrophic illness” and are hence exempt from copayment. In Taiwan, SS is diagnosed based on the classification criteria for SS proposed by the American-European Consensus Group in 2002 [13]. Patients are issued the certificate of catastrophic illness for SS if their SS diagnosis is validated by two qualified rheumatologists after athorough review of patients’ medical records, laboratory data, and images. The NHIRD also contains a catastrophic illness enrollment file forpatients with catastrophic illness certificates, namely the Registry for Catastrophic Illness Patient Database (RCIPD). In the present study, SS patients were included only when they were found in the RCIPD. Additionally, the NHIRD constructed a representative database of 1,000,000 individuals randomly selected from all enrollees who received services in 2000 (Longitudinal Health Insurance Database, LHID2000). In this study, the data of the non-SS control group were extracted via matching SS cases for age, sex, and the year of first SS diagnosis date from this LHID 2000 database.

### Study samples

#### Incident SS cases identified from whole Taiwanese population

SS patients were defined as having at least three ambulatory visits or one hospital admission with a diagnosis of SS [International Classification of Diseases, 9^th^ Revision, Clinical Modification (ICD-9-CM) code 710.2] and a catastrophic illness certificate for SS. From the NHIRD, after exclusion of individuals who ever had a diagnosis of rheumatoid arthritis (RA) (ICD-9-CM code 714.0) or systemic lupus erythematosus (SLE) (ICD-9-CM code 710.0) during 2003–2013, we included all newly diagnosed SS cases from 2007 to 2012 as incident SS cases. The first date of visit with SS diagnosis was selected as the index date. The index year was the year of the index date.

#### Matched non-SS controls selected from representative one million populations

From LHID 2000, after exclusion of individuals who ever had ICD-9 codes for SS, RA or SLE during 2003–2013, we randomly selected non-SS controls, matching SS cases (1:15) for age, sex and the index year. Given that in non-SS controls, no diagnosis of SS was made, the index date used for non-SS controls was the day of first ambulatory visit for any reason in the index year.

#### Definitions of mycobacterial infection

Mycobacterial infection was identified by ICD-9-CM codes for TB: 010–018 and NTM: 031.0, 031.1, 031.2, 031.8, and 031.9 with the concurrent prescription of at least two anti-mycobacterial drugs within 12 months of the diagnosis. Anti-TB drug prescriptions included isoniazid, ethambutol, rifampin, pyrazinamide, streptomycin, prothionamide, kanamycin, amikacin, levofloxacin, ofloxacin, moxifloxacin, ciprofloxacin, and thioridazine [14]. Anti-NTM drugs consisted of amikacin, cefoxitin, ciprofloxacin, clarithromycin, doxycycline, ethambutol, imipenem, levofloxacin, meropenem, minocycline, moxifloxacin, rifabutin, rifampin, tigecycline, and streptomycin [15]. When TB and NTM infections were identified concurrently based on above definitions, we refer the diagnosis as TB if pyrazinamide was prescribed for longer than 28 days and as NTM if clarithromycin was prescribed for longer than 28 days; these criteria were based on the fact that these two drugs havemutually exclusive, condition-specific indications in TB and NTM.

#### Comorbidities as potential confounders

The presence of comorbiditywas defined as having at least three ambulatory visits or one inpatient visit with a corresponding ICD-9CM code within one year before the index date. The Charlson Comorbidity Index (CCI), as adapted by Deyo *et al*. [16], was used to assess the level of general comorbid conditions. Given that bronchiectasis appeared to be correlated with both SS and mycobacterial infection [17–19], we also included bronchiectasis, as indicated by the ICD-9-CM code 494, as a potential confounder.

#### Statistical analysis

The demographic data were presented as a mean ± standard deviation for continuous variables and as a percentage of patients in categorical variables. The differences were analyzed by using Student’s t-test for continuous variables and Pearson’s χ2 test for categorical variables. The history of mycobacterial infection in SS compared with those in controls was shown as an adjusted odds ratio (aOR) using multivariate logistical regression analysis after adjusting for CCI and the diagnosis of bronchiectasis. The regressionwas further stratified by age, gender, CCI, and bronchiectasis. The significance of modification effect by the covariate used to stratify patients for TB or NTM infection-associated SS risk was investigated by estimating the p-value of the coefficient associated with the product of each indicator of the covariate and the indicator of TB or NTM infection using the Wald test. All data was analyzed using statistical software version 9.3 (SAS Institute, Inc., Cary, NC, USA). A p-value <0.05 was considered statistically significant.

## Results

### Characteristics of the study population

A total of 5,751 SS cases and 86,265 matched non-SS controls were assessed (see S1 Dataset for details).We found that SS cases had a higher CCI (0.5±0.9vs. 0.4±1.0, p<0.001) and were more likely to have bronchiectasis (4.1% vs. 1.3%, p<0.001), TB infection (1.0% vs. 0.5%, P<0.001) and NTM infection (0.1% vs. <0.1%, p< 0.001) compared with controls (Table 1). Additionally, the mean interval between diagnosis of NTM and TB in SS group was 2.1±2.2 and 3.4±2.5 years respectively, while those in control group was 2.9±1.4 and 3.8±2.4 years respectively.

**Table 1:** Demographic data and clinical characteristics of patients.

	Non-SS controls	SS cases	
	(*n* = 86,265)	(*n* = 5,751)	p-value
**Age, years**	55±14	55±14	1
**Age group**			
≦40 years	12,945 (15.0)	863 (15.0)	
40–65 years	51,660 (59.9)	3,444 (59.9)	
≧65 years	21,660 (25.1)	1,444 (25.1)	
**Gender**			1
Female	75,765 (87.8)	5,051 (87.8)	
Male	10,500 (12.2)	700 (12.2)	
**CCI**	0.4±1.0	0.5±0.9	<0.001
**CCI group**			
0	66,918 (77.6)	3,848 (66.9)	
≧1	19,347 (22.4)	1,903 (33.1)	
**Bronchiectasis**	1,157(1.3)	235 (4.1)	<0.001
**Mycobacterial infection**	468 (0.5)	61 (1.1)	<0.001
**NTM infection**	3 (0.0)	4 (0.1)	<0.001
Interval between NTM diagnosis and SS, years	2.9±1.4	2.1±2.2	0.601
**TB infection**	465 (0.5)	57 (1.0)	<0.001
Interval between TB diagnosis and SS, years	3.8±2.4	3.4±2.5	0.233

Abbreviations: SS,Sjögren’s Syndrome; CCI,Charlson comorbidity index; NTM,nontuberculous mycobacteria; TB, tuberculosis; NA, non-applicable.Percentages are enclosed in parentheses after each value unless otherwise indicated.

### Association between a history of mycobacteria infection and the risk of Sjögren’s syndrome

A conditional logistic regression model, adjusted for CCI and bronchiectasis, found that, compared with non-SS controls, SS cases were more likely to have a history of mycobacterial infection (aOR 1.36, 95% CI 1.03–1.80), diagnosis of bronchiectasis (aOR 2.74, 95% CI 2.36–3.18), and CCI ≥1 (aOR 1.83, 95% CI 1.71–1.94) (Table 2). To address the distinct association of SS withTB or NTM infection, we hence classified mycobacterial infections as either TB or NTM infection, and found a markedly high association between a history of NTM infection and the risk of SS (aOR 11.24, 95% CI 2.37–53.24), whereas the correlation between a history of TB infection and SS risk did not reach statistical significance (aOR 1.29, 95% CI 0.97–1.71).

**Table 2:** Unadjusted and adjusted odds ratios for the association between variables and the risk of Sjogren's syndrome.

	Univariate analysis	Multivariable A^a^	Multivariable B^a^
	OR (95% CI)	aOR (95% CI)	aOR (95% CI)
**Mycobacterial infection**	1.98 (1.51–2.59)	1.36 (1.03–1.80)	
**NTM infection**	20.00 (4.48–89.36)		11.24 (2.37–53.24)
**TB infection**	1.86 (1.41–2.45)		1.29 (0.97–1.71)
**CCI≥1**	1.89 (1.77–2.01)	1.83 (1.71–1.95)	1.83 (1.71–1.94)
**Bronchiectasis**	3.19 (2.76–3.69)	2.74 (2.37–3.18)	2.74 (2.36–3.18)

^a^NTM and TB infection were integrated into the mycobacterial infection in model A and seen as independent variables in model B. Abbreviations:OR, odds ratio; CI: confidence intervals; NTM, nontuberculous mycobacteria; TB, tuberculosis; CCI, Charlson comorbidity index.

### Subgroup analyses

In subgroup analyses, the association between the risk of SS and a history of mycobacteria infection, TB infection or NTM infection remained statistically significant among patients aged between 40 and 65 years, female patients, those with CCI = 0, and those without bronchiectasis (Table 3). Of note, the association between NTM infection and SS risk was strongest among those aged 40–65 years (aOR 39.24, 95% CI 3.97–378.75), followed by those without bronchiectasis (aOR 37.98, 95% CI 3.83–376.92).

**Table 3:** Stratified analyses for the association between mycobacterial infection and risk of Sjogren's syndrome.

	Mycobacterial infection		TB infection		NTM infection	
	OR (95%CI)	p*	OR (95%CI)	p*	OR (95%CI)	p*
**Age group**		0.085		0.140		0.092
≤40 years	2.08 (0.77–5.61)		2.08 (0.77–5.61)		—	
40–65 years	1.81 (1.21–2.70)		1.62 (1.07–2.47)		39.24 (3.97–387.75)	
≥65 years	1.02 (0.67–1.56)		1.00 (0.65–1.54)		2.45 (0.22–27.22)	
**Gender**		0.342		0.481		—
Female	1.51 (1.10–2.08)		1.39 (1.00–1.94)		11.41 (2.42–53.93)	
Male	0.99 (0.56–1.74)		0.99 (0.56–1.74)		—	
**CCI group**		0.008		0.013		—
0	2.26 (1.46–3.47)		2.07 (1.33–3.24)		>999 (—)	
≥1	1.09 (0.72–1.64)		1.04 (0.68–1.59)		4.17 (0.36–48.98)	
**Bronchiectasis**		0.072		0.097		0.092
No	1.62 (1.18–2.22)		1.51 (1.09–2.10)		37.98 (3.83–376.92)	
Yes	2.63 (0.79–8.76)		2.35 (0.69–7.97)		>999 (—)	

*p for interaction. Abbreviations: NTM: nontuberculous mycobacteria; TB: tuberculosis; CCI: Charlson comorbidity index.

### Long treatment course in subjects with NTM infection

Table 4 details the medication history in subjects with NTM infection. As expected, all of the seven subjects with NTM infection underwent the clarithromycin-containing regimen, except for one subject (subject 4) who might be intolerant of clarithromycin and was treated mainly with moxifloxacin and doxycycline (Table 4). The treatment duration was quite long in subjects with NTM infection, with 341.0±130.8 days. Taken together, these data support the idea that the identified patients with NTM infection in this study were notmerely NTM colonization.

**Table 4:** Age and number of days of prescription anti-mycobacterial antibiotics given to the seven subjects with NTM infection.

	Age	Overall	Clarithromycin	Ethambutol	Rifampin	Isoniazid	Moxifloxacin	Doxycycline
**Subjects with SS**								
Subject 1	64	366	366	366	366			
Subject 2	78	455	427	329	455	455		
Subject 3	42	490	154	490	490	490		
Subject 4^a^	46	369	29	21	21	21	369	109
**Subjects without SS**								
Subject 1	71	381	381	381	381			
Subject 2	88	186	186	186	186			
Subject 3	58	140	140	28	28	28	14	

^a^Also received cefoxitin and rifabutin for 25 days. Abbreviations: NTM: nontuberculous mycobacteria; SS: Sjögren’s Syndrome.

## Discussion

To our knowledge, the present study is the first to investigate the association between mycobacterial infection andthe risk of SS using a population-based dataset. The present study demonstrates an association between NTM infection and incident SS. Such association was remarkably high among subjected aged 40–65 years and those without bronchiectasis. Our results suggest the need for surveillance for SS in NTM infected patients, especially those who are 40–65 years of age and who did not have bronchiectasis, so as to diagnose and treat SS early. Possible explanations for the high association between NTMinfection and SS riskas shown in this study include similar immune pathways, mycobacterium-initiated autoimmunity and reverse causality due to the insidious progression of SS.

First, both SS and NTM infections are apparently to occur in postmenopausal women, indicating potential shared immune mechanismsand a possible role of sex hormones in these two diseases [20–22]. Mavragani *et al*. reported increased circulating type I interferon (IFN) levels with upregulated type I IFN-inducible genes in salivary gland tissues of patients with SS [20]. Type I IFN has also been found recently to play a major role in the dysregulated inflammation of mycobacterial infection [23], making it a potential target for immune therapy, so-called host-directed therapy, aiming to alleviate the host-detrimental inflammation in mycobacterial infection [24]. Upregulated type I IFN may thus be one example of shared immunological pathways in these two highly correlated diseases. Furthermore, a low level of serum sex hormone, namely dehydroepiandrosterone (DHEA), has been identified in women with pulmonary *Mycobacterium avium complex* infection [25], while a lack of estrogen and DHEA has been found to result in increased apoptosis of the exocrine secretory cells in SS [21]. These mechanisms may at least partly explain the high correlation between NTM infection and SS development in postmenopausal women.

Second, dysregulated inflammation against aninert and relatively low-virulence mycobacteriumhas been found to play a crucial role in mycobacterial infections [26]. Autoimmunity has similarly been proposed to correlate with a dysregulated, non-resolving, and host-detrimental inflammation in mycobacterial infection [7, 27]. Some findings with regards to mycobacterium-related autoimmunity have been reported, including that anti-cyclic citrullinatedpeptide antibody was found in 37% of TB patients [28], and the anti-IFN-gamma autoantibody in subjects with disseminated NTM infection [29]. Sarcoidosis, an autoimmune disease without known autoantigen, maybe another piece of evidence linking autoimmunity and mycobacteria, because of the indistinguishable granulomatous inflammation common to both sarcoidosis and mycobacterial infection [30]. Heat-shock protein has been implicatedin the initiation of autoimmunity in TB infection [7]; however, the crucial causal factor to initiate autoimmunity in mycobacterial infection remains elusive at this point. In this study, we found a much higher correlation between NTM and SS than those between TB and SS; this finding might be explained by the high probability of exposure to environmental NTM and further support the possibility of infection-induced autoimmunity in NTM infection.

Third, SS is a disease with insidious progression [2, 3] and has an increased risk of infection including TB [10]. In this study, of the seven subjects with NTM infection and later diagnosed SS, three of them were diagnosed as SS within three months after NTM infection, indicating the potential co-existence of these two diseases, while the other four subjects were diagnosed as SS averagely 2.9 years after NTM infection. Therefore, among SS cases with a history of NTM infection, we cannot exclude the possibility that SS had developed before NTM infection. However, the significant association between NTM infection and newly diagnosed SS demonstrated in our study may suggest a need to survey the existence of SS for NTM infected patients.

Although the association between TB infection and SS risk did not reach statistical significance (aOR, 1.29; 95% CI, 0.97–1.71) among all study subjects, such association was significant in those with CCI = 0 (aOR, 2.07; 95% CI, 1.33–3.24). Of note, the modification effect by CCI for this association was also statistically significant (p-value for interaction = 0.013). Therefore, this finding suggests that mycobacterial infection might still be a risk factor for incident SS among individuals without any comorbidities used for CCI calculation.

There are limitations in this study that merit discussion. First, information of NTM species was lacking in this claim-based dataset. Therefore, we cannot assess the association between SS risk and specific NTM species in this study.However, we thought the data of this study merits further mechanistic studies. Second, given the fact thatRA and SLE are associated with mycobacterial infection and SS [15], this study excluded patients with RA or SLE; therefore, the results cannot be applied to patients with RA- or SLE-related secondary SS. Third, the accuracy of diagnoses according to claims data is of concern. However, the regular check of the quality of claims data from all medical institutions by the BNHI has improved coding accuracy [31] and thus minimized bias due to misclassification. Also, the accuracy of SS diagnosis is of less concern since at least two qualified rheumatologists had validated the diagnosis before SS cases were issued a catastrophic illness certificate. The non-differential misclassification bias related to TB or NTM diagnosis in both groups is always biased toward the null, leading to underestimating the association of SS risk with TB or NTM. The prolonged study period 2007–2012 could be another concern; however, the diagnosis and treatment was consistent during the study period. Briefly, Taiwan Center for Disease Control (CDC) has conducted the external quality assessment of clinical mycobacteriology laboratories since 2007 [32], and the treatment of TB in Taiwan has been standardized since 2005 with directly observed therapy-short course (DOTS) by Taiwan CDC [33]. Additionally, in subjects with NTM infection, the average duration of clarithromycin-use was 276±129 days, and such a long-course clarithromycin-contained regimen was in accordance with the NTM treatment guideline published in 1997 and the revised edition published in 2007 [34, 35].

## Conclusion

In conclusion, using a nationwide, population-based dataset, this study revealed a significant association between NTM infection and the risk of SS, in particular among those aged 40–65 years and those without a history of bronchiectasis. We believed that these data mightsupport the need for screening for SS in patients with NTM infection, particularly among those aged 40–65 years and those without a history of bronchiectasis. More studies are warranted in the future to explore underlying mechanisms.

## Supporting information

S1 Dataset Data of the SS cases and non-SS controls. (XLSX)

## Abbreviations

BNHI: bureau of national health insurance
CI: confidence intervals
CCI: Charlson comorbidity index
DHEA: dehydroepiandrosterone
ICD: international classification of diseases
IFN: interferon
LHID: longitudinal health insurance database
NHI: national health insurance
NHIRD: national health insurance research data
NTM: nontuberculous mycobacterium
OR: odds ratios
RA: rheumatoid arthritis
RCIPD: registry for catastrophic illness patient database
SLE: systemic lupus erythematosus
SS: Sjögren’s syndrome
TB: tuberculosis
